# Semen quality in Peruvian pesticide applicators: association between urinary organophosphate metabolites and semen parameters

**DOI:** 10.1186/1476-069X-7-59

**Published:** 2008-11-17

**Authors:** Sandra Yucra, Manuel Gasco, Julio Rubio, Gustavo F Gonzales

**Affiliations:** 1 Department of Biological and Physiological Sciences, Faculty of Sciences and Philosophy, Universidad Peruana Cayetano Heredia, Lima, Peru; 2 Instituto de Investigaciones de la Altura, Universidad Peruana Cayetano Heredia, P.O. Box 1843, Lima, Peru

## Abstract

**Background:**

Organophosphates are broad class of chemicals widely used as pesticides throughout the world. We performed a cross-sectional study of associations between dialkylphosphate metabolites of organophosphates and semen quality among pesticide applicators in Majes (Arequipa), Peru.

**Methods:**

Thirty-one men exposed to organophosphate (OP) pesticides and 31 non-exposed were recruited (age, 20–60 years). In exposed subjects, semen and a blood sample were obtained one day after the last pesticide application. Subjects were grouped according to levels of OP metabolites in urine. Semen samples were analyzed for sperm concentration, percentage of sperm motility, percentage of normal morphology, semen leucocytes and concentrations of fructose and zinc. Exposure to OP was assessed by measuring six urinary OP metabolites (dimethyl and diethyl phosphates and thiophosphates) by gas chromatography using a single flame photometric detector.

**Results:**

Diethyldithiophosphate (p = 0.04) and diethylthiophosphate (p = 0.02) better reflected occupational pesticide exposure than other OP metabolites. Semen analysis revealed a significant reduction of semen volume and an increase in semen pH in men with OP metabolites. Multiple regression analysis showed that both occupational exposure to pesticides and the time of exposure to pesticides were more closely related to alterations in semen quality parameters than the single measurement of OP metabolites in urine.

**Conclusion:**

The study demonstrated that occupational exposure to OP pesticides was more closely related to alterations in semen quality than a single measurement of urine OP metabolites. Current measurement of OP metabolites in urine may not reflect the full risk.

## Background

A wide variety of synthetic chemicals are found in the environment as a consequence of modern manufacturing processes and agricultural productivity efforts [1]. Agriculture is an important activity and source of economic income in many parts of the world, especially in developing countries.

Organophosphate (OP) pesticides are the most commonly used pesticides in Peruvian agriculture. The population at risk for OP exposure includes formulators, applicators and farmers [2]. One of the problems in the use of this kind of pesticides is the inadequate protection during pesticide application [2,3].

Exposure to OP causes neurologic dysfunctions that include central and peripheral neurologic symptoms [4-8]. Also, these chemicals have numerous other compound specific effects, including those occurring in the reproductive system [4,9,10].

However, there are conflicting results on the effects that OP exposure has on semen quality. Emerging evidence suggests that it may reduce semen quality in exposed workers [6,7,5,11].

Sanchéz-Peña et al observed, in Mexican agricultural workers, that OP exposure alters sperm chromatin condensation, increasing the number of cells with greater susceptibility to DNA denaturalization [12]. Other authors suggested that OP exposure could interfere with sperm chromosome segregation and increase the risk for genetic syndromes [13]. However, other studies did not find any relation between changes in sperm parameters and pesticide exposure variables [14,15]. Differences seem to be due, at least in part, to the quality of protective measures when facing OP pesticides exposure.

Studies on serum hormone levels are also conflicting [16-18,14,15]. Experimental studies in laboratory animals described an anti-androgenic activity for two OP pesticides: Fenitrothion [19] and Chlorpyrifos-methyl [20].

Peruvian pesticide sprayer applicators in Majes, Arequipa, had significantly reduced age-adjusted seminal volume, percentage of motility, percentage of sperm with normal morphology, serum luteinizing hormone, serum testosterone levels, and seminal zinc concentration (a marker of prostate function). They also showed significantly increased time of liquefaction, seminal pH, percentage of immature sperm morphology, and leukocyte concentration. Use of pesticide sprayers were characterized by inadequate practice of safety measures [2]. These findings provided evidence that occupational exposure to OP pesticides adversely affects semen quality and sex hormones [11].

However, OP metabolites were not measured in this study. In order to determine the association, if any, between OP and semen quality, we attempted to correlate seminal quality with three ethylated and three methylated OP metabolites measured in the urine of pesticide sprayer users from the Majes valley, a farm region in southern Peru. Concentrations of urine OP metabolites were related to each semen parameter.

## Methods

### Study design

A cross-sectional study was performed, based on interviews and collection of semen and blood samples from 31 OP pesticide applicators (exposed group) and 31 non-exposed individuals. Inclusion criteria for subjects of the exposed group included history of work with pesticides and length of residence in Majes for at least two years prior to the study. The age of subjects ranged from 20 to 60 years old. Subjects in control group were included in the study if they had never worked as pesticide applicators and were not currently exposed to pesticides, either occupationally or non-occupationally.

Subjects in both groups were not sick at the time of the study; they had not taken any medication for at least three months prior to the study and had lived at least two years in Majes.

Regarding control group selection, some exposed subjects were asked to recruit a male friend without any exposure to OP or agricultural activity. Also, healthy men who responded to an announcement at the medical center, hotel, or municipality were invited to participate in the study. Subjects from the medical center were staff members. All subjects in the non-exposed group had lived at least two years in Majes.

The study was approved by the Institutional Review Board (IRB) of Universidad Peruana Cayetano Heredia, Lima-Peru. A signed informed consent was obtained from each study participant.

### Study area

Majes is an agricultural area located in Caylloma, Arequipa. It is one of the main agricultural production areas in the Southern part of Peru. It is situated at 1420 m. above sea level. Its temperate climate makes agricultural production possible almost all year round. OP pesticides are used on a variety of crops including potatoes, alfalfa, onions, tomatoes, garlic, apples and grapes. Methamidophos is the most frequently used OP pesticide in the Majes valley [2].

### Recruitment of study population

Pesticide applicators eligible for participating in the study were identified and recruited by agronomic engineers working in Majes Valley. From the universe of applicators in Majes (150 pesticide applicators), 64 accepted to participate in the study. Of these, 31 fulfilled the inclusion criteria, which were: i) To be working as a pesticide applicator for at least 2 years; ii) To have used pesticides within a week before the questionnaire application and semen sample analysis. Data from questionnaire, and blood and semen samples were collected one day after the last pesticide use reported.

### Questionnaire

The questionnaire was administered to each pesticide applicator to obtain information on sociodemographic characteristics; agricultural work practices, and knowledge and practice of safety guidelines for pesticide use.

Applicators were asked to define how frequently they use OP pesticides. Data related to the kind of pesticides used, protective measures practiced during application, and management of pesticides and clothes after pesticide application were also recorded.

### Semen collection and Analysis

All participating subjects were asked to abstain from ejaculation for 3 to 5 days before semen collection. Information on date, time, spillage, occurrence of fever, and number of days since last ejaculation was recorded for each sample. The semen was collected by masturbation in a private room and analyzed on-site within one hour for both macroscopic and microscopic characteristics. Also, seminal fructose, true-corrected fructose, and zinc levels were determined [21].

Semen analysis was performed according to the protocol described in the WHO laboratory manual for the examination of human semen and sperm-cervical mucus interaction. Semen analysis included: liquefaction time, seminal volume, pH, sperm concentration, total sperm number (sperm concentration × seminal volume), sperm morphology, sperm motility, sperm viability and concentration of leukocyte determined by peroxidase staining using orthotoluidine blue method [22]. A Neubauer improved bright-line hematocytometer (Marienfeld Laboratories, Germany) was used to determine sperm and leukocyte concentrations.

Time of liquefaction was measured as the time in minutes it took for the semen specimen to liquefy. Fresh ejaculated human semen is coagulated, however, due to proteolytic enzymes present in the prostatic secretions, the semen will liquefy. If liquefaction is not complete, the examiner may observe gel particles or mucous streaks.

According to the WHO, a semen sample liquefies within 15 minutes at room temperature however the normal range extends to 60 minutes [22]

Volume was also assessed macroscopically. The volume of ejaculate was measured in milliliters using a graduated cylinder. The normal or reference value for volume of ejaculate is considered to be 2 milliliters or more by WHO standards [22].

Morphology describes the shape of the sperm (including the head, neck, mid-piece and tail) and is expressed as the percentage of sperm that meet "normal" morphology criteria defined by WHO. Sperm morphology was evaluated microscopically. A smear from the fresh semen sample was stained with Giemsa in order to view and count the number of normal and abnormal spermatozoa until at least 200 consecutive spermatozoa were evaluated. Generally, the normal value for sperm morphology is at least 30%. No strict criteria were used for the assessment of sperm morphology.

Viability measures the percentage of sperm that are alive because it is possible that sperm may be alive but not moving. Sperm viability was assessed using an Eosin dye. Under the microscope, the examiner was able to differentiate the live (unstained) sperm from the dead (stained) sperm and calculate the percentage of viable sperm. The reference value for sperm viability is 75% [22]. Sperm motility was also analyzed microscopically. Motility describes the percentage of sperm that are moving.

Using an unstained sample of fresh semen, the number of motile sperm was counted until a total of 200 spermatozoa were assessed. The procedure was conducted twice to improve accuracy of the measurement. Motility is classified in four categories:

(a) If sperm has a rapid and linear movement,

(b) If sperm has a slow or sluggish linear or non-linear movement,

(c) If it has a non-progressive motility,

(d) If sperm is immotile,

According to the WHO, 50% or greater motility grades a and b or 25% or greater sperms grade a are considered normal [22]. Sperm concentration or density, sometimes referred to as the "count," is a measurement of millions of sperm per milliliter. By looking at a diluted semen sample under the microscope, the examiner counted the number of spermatozoa in a defined field of view. The procedure was repeated to ensure accuracy and a conversion factor was used to calculate the concentration. The normal sperm concentration value is at least 20 million spermatozoa per milliliter [22]

Knowing the sperm concentration and volume, the total number of sperm in the ejaculation was calculated (sperm concentration × volume = total number of sperm). Total number of sperm is expressed in millions of spermatozoa per ejaculation. The reference value for total sperm number is at least 40 million spermatozoa per ejaculation [22].

Additionally, the concentration of zinc in seminal plasma was determined by spectrophotometric method using a commercial kit (RANDOX Laboratories, United Kingdom). The proteins in the sample were precipitated with trichloroacetic acid, the supernatant mixed with a water-soluble pyridylazo dye and the absorbance measured at 560 nm. Also, fructose was measured in semen using a spectrophotometric method.

True-corrected fructose is calculated by multiplying the log of motile sperm concentration by the seminal fructose concentration [23]. Although true-corrected fructose is not included in the WHO manual, previous studies have demonstrated that true-corrected fructose is a better marker of seminal vesicle function [24].

Fructose is a compound secreted by the seminal vesicles. Measurement of seminal fructose used universally as a marker of the seminal vesicle function is not an appropriate approach due to its inverse relationship with the sperm count. The true corrected fructose defined as [log. motile sperm concentration] multiplied by [seminal fructose concentration] has been shown to be a better marker of the seminal vesicle function [25].

Zinc is a marker of prostate function [11]. Low zinc concentration associated to high pH is associated to low prostatic secretion [21].

Leukocytes in semen were measured with peroxidase stain using ortholuidine blue as suggested by WHO. The percentage of peroxidase positive neutrophiles was recorded. Leukocytospermia is diagnosed if semen sample has more than 1 million leucocytes peroxidase positive [22]. All seminal analyses were performed in duplicate by the same investigator who did not know about the characteristics of the subjects.

### Blood collection and reproductive hormones assay

Venous blood samples were obtained after a 12-h overnight fast. Blood was centrifuged at 1000 g, and serum was collected after centrifugation and kept frozen until assayed for serum testosterone (T), estradiol (E_2_), follicle stimulating hormone (FSH), and luteinizing hormone (LH) concentrations (Diagnostic Products Co., Los Angeles, CA, USA).

T and E_2 _concentrations were determined by radioimmunoassay (RIA) using 125I-labeled testosterone and 125I-labeled estradiol, respectively, as radioactive markers (Diagnostic Products Co., Los Angeles, CA, USA).

The assays were performed using commercially available kits (Diagnostic Products Co., Los Angeles, CA, USA). All samples were run in the same assay. The within-assay variation was 5.5% for testosterone and 6.4% for estradiol. The level of detection of the testosterone and estradiol assays was 0.04 ng/ml and 8.0 pg/ml respectively.

Serum FSH and LH levels were measured by immunoradiometric assay (IRMA) in solid phase using commercially available kits (Diagnostic Products Co, Los Angeles, CA, USA). Within-assay variation was 2.9% for FSH and 1.3% for LH. The sensitivity of the assays was 0.06 mIU/ml for FSH, and 0.15 mIU/ml for LH.

### Urine collection, storage and organophosphate determination

One day after applying OP pesticides, each worker was provided with one polyethylene urine collection bottle and instructed to collect a urine sample. For this purpose, the first avoid in the morning was collected. All the collected urine samples were immediately placed inside a plastic container with ice and transported to the medical center for freezing at -20°C. The time between urine collection and freezing was 10–15 minutes.

After collection was completed, all samples were shipped frozen to Pacific Toxicology Laboratories (Los Angeles, California U.S.A) where the following metabolites of organophosphates were measured: Dimethylphosphate (DMP), Dimethylthiophosphate (DMTP), Dimethyldithiophosphate (DMDTP), Diethylphosphate (DEP), Diethylthiophosphate (DETP) and Diethyldithiophosphate (DEDTP). All urine samples were stored at -20°C until extraction. Urine pH was not adjusted prior to freezing.-20°C

The standards of DMP (100% purity), DMTP (99% purity), DMDTP (98% purity), DEP (98.3% purity), DETP (99% purity), and DEDTP (99% purity) were obtained from (Cerilliant Corporation, TX, USA).

For extraction, freeze-dried urine samples were treated with a benzyltolytriazine reagent (Sigma-Aldrich Inc., Steinheim, Germany) to produce benzyl derivatives of alkylphosphate metabolites. A saturated salt solution was added to the tubes and the benzyl derivatives were extracted with cyclohexane (Sigma-Aldrich Inc., Steinheim, Germany) and analyzed by gas chromatography with flame photometric detection [26].

Likewise, the quality control was made in-house by spiking normal urine sample. We run 2 levels of in-house made urine controls. The assay was run with a reagent water blank and urine blank. The recovery rate ranged from 80 to 120% of expected value. R2 (Coefficient of determination) correlation data of methylated and ethylated samples were: (DMP = 0.99), (DMTP = 0.99), (DMDTP = 0.99), (DEP = 0.99), (DETP = 0.99), and (DEDTP = 0.99).

The limit of detection was 5 μg/l for DMP, DEP, DETP and DMTP, and 10 μg/l for DEDTP and DMDTP. Creatinine was also measured in the urine samples by a colorimetric method (Creatinine Procedure No 555; Sigma Diagnostics, St Louis, Mo). Its measurement was used to adjust results of OP metabolites (ug/gram creatinine) to avoid the variable dilution caused by the different hydration states of the sample donor.

### Data analysis

Data recorded in the questionnaires, semen and serum samples were introduced in an Excel database. Statistical analysis was performed using the statistical package STATA (version 8.0) for personal computer (Stata Corporation, TX, USA). Descriptive data were presented as mean ± standard deviation (SD), as well as frequencies. The percentage of subjects with detected OP metabolites in urine (percentage of samples above detection limit for each analyte) was also calculated. Many urine samples had concentrations below detection limits of some metabolites.

For the dialkylphosphates metabolites, the samples below the respective limit of detection (LOD) were assigned to have concentrations equal to one-half the LOD for statistical analyses as used by others in a previous report [27].

The influence of the variables was evaluated for single alkylphosphates and for the sum of dimethyl (DMP + DMTP + DMDTP), diethyl (DEP + DETP + DEDTP). We named these sums methylated, and ethylated OP metabolites, respectively. To calculate these sums, analytes below the detection limit were counted as a value half the detection limit.

Statistical analysis of the samples was then carried out, including a value half the detection limit for non detectable analytes. We used the Kolmogorov-Smirnov test to check the distribution of samples for the six alkylphosphates; we found a positive asymmetric distribution, which became normal after log transformation. Parametric analysis (multiple regressions) was therefore used for subsequent comparisons. Statistical significance was set at P < 0.05.

Homogeneity of variances was assessed using the Barlett test. Variables that were not normally distributed (corrected fructose, seminal zinc, sperm/ml, sperm/ejaculum, motility grade a, a+b, seminal leukocytes, serum levels of E_2_, FSH, LH, T/E2 and T/LH) were transformed. Student's t test was used to compare the mean between groups.

Multivariable regression analyses were performed to explore the relationship between the following dependent variables: OP metabolites levels (methylated and ethylated), time of exposure (hours worked as a pesticide applicator) and toxicity (related to the severity degree of OP pesticide used) with respect to each of the semen parameters or hormone levels as dependent variables. Each multivariate regression analyses were controlled for age and alcohol consumption. One problem with multiple comparisons is that the greater the number of tests, the higher the likelihood of falsely rejecting the null hypothesis. For this, we have used the Holm's test for correcting multiple comparisons.

## Results

Data in Table 1 show that DEDTP (p = 0.04) and DETP (p = 0.02) were more related to occupational exposure than other OP metabolites. In fact, DEP, DMP, DMDTP and DMTP were present in the same proportion in exposed and non-exposed men.

**Table 1:** Chi-square test to evaluate the presence/absence of OPs metabolites in men from Majes, Arequipa.

Dependent variable	Non Exposed n/N (%)	Exposed n/N (%)	p	No Exposed GM((DS(GM)))	Exposed GM((DS(GM))	p
DEP	5/31 (16.1)	8/31 (25.8)	0.53	2.99(2.06)	3.75 (8.17)	0.31
DEDTP	9/31 (29.0)	18/31 (58.1)	0.04	10.24(54.46)	25.33 (78.60)	0.01
DETP	2/31 (6.5)	10/31 (32.3)	0.02	2.66(1.02)	3.85 (7.15)	0.10
DMP	23/31 (74.2)	26/31 (86.9)	0.53	9.57(19.47)	14.22 (42.42)	0.14
DMDTP	5/31 (16.1)	1/31 (3.2)	0.19	56.37(6.65)	5.3 (4.53)	0.10
DMTP	18/31 (58.1)	25/31 (80.6)	0.09	9.69(68.72)	22.92 (48.55)	0.02

n = number of subjects with the metabolite in urine. N = number of subjects in each group (exposed or non exposed subjects). P: level of significance after chi square test.

LOD (Limit of detection) for: DMP, DMTP, DEP, DETP 5 ug/l. DMDTP, DEDTP 10 ug/l.

Men with ethylated OP metabolites in their urine had lower seminal volume than those without ethylated OP metabolites (p = 0.04). Men with methylated OP metabolites had higher seminal pH than men without detected methylated OP metabolites (p = 0.01). Furthermore, high levels of ethylated metabolites of OP were related to low seminal volume (p = 0.02) and high levels of methylated metabolites of OP were related to high seminal pH (p = 0.02) (data not shown).

After controlling for urine levels of ethylated OP metabolites, the exposure to pesticides (positive or negative) was related to low seminal volume (p = 0.03), high seminal pH (p = 0.01), low percent of sperm with normal morphology (p = 0.00), and high concentration of seminal leucocytes (p = 0.00) (Table 2). However, when Holm's test is applied to adjust P value, it was observed that seminal pH (p = 0.02) is the only variable associated to exposure to ethylated OP metabolites.

**Table 2:** Regression coefficients and probability values to determine the association between seminal parameters and exposure and time of exposure to ethylated OP metabolites.

	**Exposure to pesticides (β ± SEM)**	**P**	P* adjusted	**Time of exposure to pesticides (β ± SEM)**	**P**	P* adjusted
Time of liquefaction minutes	-1.53 ± 1.74	0.38	0.75	0.11 ± 0.31	0.71	0.93
Seminal Volume mL	-0.72 ± 0.31	0.02	0.12	-0.09 ± 0.06	0.15	0.28
pH	0.29 ± 0.09	0.002	0.002	0.03 ± 0.02	0.07	0.001
Log Corrected fructose	-0.17 ± 0.09	0.06	0.15	-0.04 ± 0.01	0.01	0.53
Log Seminal zinc	-0.05 ± 0.09	0.58	0.30	-0.02 ± 0.02	0.26	0.38
Log sperms/mL	-0.14 ± 0.12	0.25	0.84	-0.02 ± 0.02	0.44	0.89
Log sperms/ejaculum	-0.24 ± 0.15	0.10	0.60	-0.04 ± 0.03	0.15	0.80
Sperm Vitality %	2.07 ± 1.79	0.25	0.06	-0.21 ± 0.32	0.51	0.13
Log motility grade 3 (%)	-0.02 ± 0.09	0.78	0.71	-0.02 ± 0.02	0.14	0.82
Log motility grade 3 + 2 (%)	-0.05 ± 0.05	0.32	1.00	-0.02 ± 0.01	0.13	0.74
Sperm with normal morphology (%)	-7.65 ± 2.16	0.001	0.76	-1.04 ± 0.42	0.01	0.42
Immature sperm (%)	1.89 ± 1.48	0.21	1.00	0.17 ± 0.26	0.51	0.50
Log Seminal leucocytes	0.59 ± 0.09	0.00	1.00	0.02 ± 0.02	0.36	0.97
Serum testosterone (ng/mL)	-0.62 ± 0.41	0.13	0.69	-0.10 ± 0.07	0.16	0.99
Log Serum estradiol (pg/ml)	0.06 ± 0.07	0.43	0.26	0.03 ± 0.01	0.02	0.65
Log Serum FSH (mIU/mL)	0.17 ± 0.18	0.34	0.86	0.01 ± 0.03	0.72	1.00
Log Serum LH (mIU/mL)	-0.37 ± 0.21	0.07	0.97	-0.10 ± 0.03	0.01	0.99
Log T/E2 (ng/pg)	-0.11 ± 0.07	0.12	0.18	-0.04 ± 0.01	0.01	0.63
Log T/LH (ng/mIU)	0.32 ± 0.20	0.13	1.00	0.01 ± 0.03	0.01	0.85

Regressions adjusted for age and alcohol consumption. Exposure to pesticide: 0 = non exposed; 1 = exposed. Ethylated metabolites of OPs: 0 = non detectable; 1 = over limit of detection. Time of exposure to pesticides (continuous variable). β ± SEM: Coefficient of regression ± Standard error of the mean. Data with non parametric behavior were log-transformed in order to performed linear regression analyses. *Holm adjustment.

+Analyses were performed after controlling for urine levels of ethylated OP metabolites, respectively.

After regressing time of exposure to pesticides (months), lower seminal vesicles function (low seminal corrected fructose levels) (p = 0.01), low percent of normal sperm morphology (p = 0.02), high serum estradiol levels (p = 0.03), low serum LH levels (p = 0.01), low ratio testosterone/estradiol (p = 0.01) and high ratio testosterone/LH (p = 0.01) were observed (Table 2). After Holm's adjustment, seminal pH values (p = 0.001) was the only variable associated to time of exposure to ethylated OP metabolites.

After controlling urine levels of methylated OP metabolites, exposure to pesticide was related to low seminal volume (p = 0.02), high seminal pH (p = 0.01), low seminal vesicles function (p = 0.05), low percent of sperm with normal morphology (p = 0.002), high concentration of seminal leucocytes (p = 0.01) and low levels of serum LH (p = 0.05) (Table 3). When Holm's test is applied to adjust P value, it was observed that seminal pH (p = 0.002) and log of corrected fructose (p = 0.04) were the only variables associated to exposure to methylated OP metabolites.

**Table 3:** Regression coefficients and probability values to determine the association between seminal parameters and exposure and time of exposure to methylated OP metabolites.

	**Exposure to Pesticides (β ± SEM)**	**P**	P* adjusted	**Time of exposure to pesticides (β ± SEM)**	**P**	P* adjusted
Time of liquefaction minutes	-1.15 ± 1.73	0.51	0.84	0.17 ± 0.30	0.56	0.95
Seminal Volume mL	-0.76 ± 0.32	0.02	0.12	-0.09 ± 0.06	0.14	0.29
pH	0.24 ± 0.09	0.01	0.002	0.03 ± 0.02	0.08	0.001
Log Corrected fructose	-0.17 ± 0.09	0.05	0.04	-0.04 ± 0.02	0.01	0.28
Log Seminal zinc	-0.05 ± 0.09	0.61	0.40	-0.03 ± 0.02	0.18	0.74
Log sperms/mL	-0.10 ± 0.12	0.43	1.00	-0.01 ± 0.02	0.51	0.76
Log sperms/ejaculum	-0.20 ± 0.12	0.171	0.5	-0.04 ± 0.03	0.12	0.84
Sperm Vitality %	2.20 ± 1.79	0.22	0.05	-0.21 ± 0.32	0.52	0.15
Log motility grade 3 (%)	-0.03 ± 0.09	0.73	0.90	-0.02 ± 0.01	0.18	1.00
Log motility grade 3 + 2 (%)	-0.05 ± 0.05	0.38	0.52	-0.01 ± 0.01	0.14	0.27
Sperm with normal morphology (%)	-7.03 ± 2.14	0.01	0.42	-0.86 ± 0.39	0.03	0.48
Immature sperm (%)	1.12 ± 1.51	0.46	1.00	0.14 ± 0.27	0.61	0.98
Log Seminal leucocytes	0.56 ± 0.09	0.01	0.68	0.02 ± 0.02	0.39	0.95
Serum testosterone (ng/mL)	-0.67 ± 0.41	0.10	0.59	-0.10 ± 0.07	0.19	1.00
Log Serum estradiol (pg/ml)	0.05 ± 0.07	0.45	0.21	0.03 ± 0.01	0.03	0.38
Log Serum FSH (mIU/mL)	-0.13 ± 0.18	0.46	0.89	0.01 ± 0.03	0.76	1.00
Log Serum LH (mIU/mL)	-0.40 ± 0.20	0.05	0.99	-0.11 ± 0.04	0.01	0.97
Log T/E2 (ng/pg)	-0.12 ± 0.07	0.11	0.18	-0.04 ± 0.01	0.01	0.56
Log T/LH (ng/mIU)	0.34 ± 0.20	0.09	1.00	0.10 ± 0.04	0.01	1.00

Regressions adjusted for age and alcohol consumption. Exposure to pesticide: 0 = non exposed; 1 = exposed. Ethylated metabolites of OPs: 0 = non detectable; 1 = over limit of detection. Time of exposure to pesticides (continuous variable). β ± SEM: Coefficient of regression ± Standard error of the mean. Data with non parametric behavior were log-transformed in order to performed linear regression analyses. *Holm's test adjustment.

+Analyses were performed after controlling for urine levels of methylated OP metabolites, respectively.

After regressing time of exposure to pesticides, high seminal pH (p = 0.05), low seminal vesicles function (low seminal corrected fructose levels) (p = 0.01), low percent of normal sperm morphology (p = 0.04), high serum estradiol levels (p = 0.02), low serum LH levels (p = 0.02), low ratio testosterone/estradiol (p = 0.01) and high ratio testosterone/LH (p = 0.02) were observed (Table 3). After Holm's adjustment, seminal pH values (p = 0.001) was the only variable associated to time of exposure to methylated OP metabolites

## Discussion

Organophosphate insecticides have been extensively used in agriculture in developing countries, with little protection for the communities and individuals thus exposed. Given the indisputable chronic exposure of vulnerable groups to organophosphate compounds, including pregnant women, the fetus and young children, the potential for widespread adverse effects is considerable [28]. As the use of these agents increases, acute and chronic exposure has become more common. As with other organophosphates, chlorpyrifos kills insects and other animals, including human beings, because of its toxicity to the nervous system [29]. Salem et al. determined the effect of chronic treatment with two sublethal doses of Dimethoate, an OP pesticide. The pesticide treatment resulted in a decline in body weight, libido, ejaculate volume, sperm concentration and semen initial fructose; and an increase in abnormal and dead sperm. The hazardous effect of these pesticides on semen quality continued during the post treatment period, and was dose-dependent [30]. Therefore Rey et. al showed that quinalphos may exert a suppressive effect on the functional activity of accessory sex glands by decreasing testicular testosterone production following inhibition of pituitary gonadotrophins release [31]. Sarkar et al probed the effect of chronic sub-lethal doses (7–14 mg kg-1 a day for 15 days) of quinalphos in rats. They found that quinalphos decreases fertility in adult male rats by affecting the pituitary gonadotrophins [32]. In another study Shigeyuki et al found that fenthion acted as antagonist of the androgenic activity of dihydrotestosterone [33].

We have previously demonstrated that several seminal parameters were affected in pesticide sprayer applicators from Majes, Peru. Seminal volume, sperm motility, normal sperm morphology, seminal zinc concentration and serum testosterone and LH were lower whereas seminal pH, time of liquefaction, percent of immature sperms and leukocyte concentration were higher in the exposed group. The multivariable analysis confirmed that seminal volume, seminal pH, sperm morphology, seminal zinc concentration and serum LH and T/LH ratio were related to exposure to pesticide and/or degree of toxicity of pesticides [11]. We found an increase of estradiol after regressing time of exposure to pesticides, this result is different from those obtained by Hodgson and Rose where OP showed a high irreversible inhibition activity of testosterone and estradiol metabolism by cytochrome P450 (CYP) 3A4, and by CYP3A4 and CYP1A2, respectively. All of these CYP inhibitions are believed to be due to the release of reactive sulfur during CYP-catalyzed oxidative desulfuration [34].

The present study was designed to demonstrate the association between parameters of seminal quality and the presence of ethylated or methylated OP metabolites in urine of men. Data did not demonstrate the association described before comparing pesticide sprayer applicators with control subjects [11,35,36]. In fact, men with elevated levels of OP metabolites in urine showed only reduction of seminal volume and elevation of seminal pH. Multivariable analysis showed that the pesticide sprayer applicator occupation and time of exposure to pesticides were more related to variations in several seminal parameters than current measurements of OP metabolites in urine.

Data suggest that current presence of OP metabolites in urine is not an adequate marker to demonstrate the effect of long-term exposure to OP pesticides. This has also been suggested in a previous study [12], since the rapid metabolism and excretion of OP and their metabolites may hinder an adequate exposure assessment. As observed in the multivariable analysis, variation in seminal quality is observed when occupational exposure to OP pesticides is included in the model. However, the fact that seminal volume and seminal pH were related to levels of OP metabolites suggested that acute exposure to dialkylphosphates pesticides could be associated to alterations in sex accessory glands.

We have grouped subjects as having ethylated or methylated metabolites since other studies found that ethylated metabolites affect sperm chromatin but not other seminal parameters [12] whereas methylated metabolites do not [37]

Seminal volume was reduced, whereas seminal pH was increased. This may be observed in situations in which the sex accessory-gland function is altered. Seminal vesicles and prostate contribute 60% and 30% of the seminal volume, respectively [38,11]. In addition, seminal vesicles contribute with basic pH and prostate with acid pH. Regarding to pH values, higher and lower values are observed in subjects with detectable levels of methylated and ethylated metabolites, respectively. These outcomes may be related to a random rather than a real biological effect of OP. For instance, if volume is affected we must also observe changes in values of zinc, fructose, and seminal pH.

One limitation with multiple comparisons is that null hypothesis may be rejected as a result of error type I. For this, we have used the Holm's test correction. Under this procedure, it may be observed that seminal pH was altered after OP exposure. In the presence of methylated OP metabolites a marker of the seminal vesicle function was also altered.

Environmental exposure to OP and adverse reproductive outcomes in men and women working on or living near farms are increasingly reported worldwide [39]. Experimental studies have demonstrated that OP pesticides, such as malathion, may induce arrest in spermatogenesis mice [40]. However, the occupational exposure [11] or the presence of either elevated levels of ethylated or methylated OP metabolites in urine suggest that occupational OP exposure seems to affect post-testicular semen rather than exert a direct action on the testis. This is based on the fact that affected parameters such as seminal volume and seminal pH may be affected independently from the testis. It is possible that the action of OP pesticides may occur at the sex accessory gland level as demonstrated in alteration at the level of seminal pH and corrected fructose, a marker of the seminal vesicles function.

Seminal vesicles and prostate function are important for chromatin stability [41]. It is also known that OP may affect DNA integrity [13,12]. If OP affects sex accessory gland functions, then, this may affect sperm DNA [42]. Effect of OP seems to be accumulative since the single measurement was unable to demonstrate all the reproductive effects observed when variable exposure to pesticides was included in the regression analysis.

In this study we found some non-exposed subjects with low levels of metabolites. These findings were similar to others where subjects considered non-exposed to OP and without relation with agricultural activities [43]. In addition, Oglobline et. al measured urinary concentrations of dialkyl metabolites in 48 adults from Australia that were not occupationally exposed to organophosphates [44]. Several types of evidence suggest that agricultural pesticide use may be an important source of non-occupational pesticide exposure [45]. Recent epidemiologic findings support the concern that non-occupational exposure to agricultural pesticides can cause acute symptoms among populations living near sprayed fields in developing countries [46]. The authors showed that one or more of the dialkyl phosphate metabolites was detected in all samples, and one sample contained all six metabolites in levels above the detection limit.

## Conclusion

The study demonstrated that occupational exposure to OP pesticides was more closely related to alterations in semen quality than a single measurement of urine OP metabolites. Current Measurement of OP metabolites in urine may not reflect the full risk.

## Abbreviations

IRMA: immunoradiometric assay; RIA: radioimmunoassay; FSH: follicle stimulating hormone; LH: luteinizing hormone; WHO: world health organization; IRB: Institutional Review Board; OP: Organophosphate; T: Testosterone; E2: estradiol; DAP: dialkylphosphates; DMP: Dimethylphosphate; DMTP: Dimethylthiophosphate; DMDTP: Dimethyldithiophosphate; DEP: Diethylphosphate; DETP: Diethylthiophosphate; DEDTP: Diethyldithiophosphate.

## Competing interests

The authors declare that they have no competing interests.

## Authors' contributions

SY was the leader of the research she contributed to all phases in the research project from conception and design, the acquisition and analysis of data and the writing of the manuscript. MG contributed to the conception and the acquisition of data. JR contributed to the analysis of data and the revising of the manuscript for intellectual content. GGR contributed to the design, the analysis of data and the revising of the manuscript for intellectual content. All authors have given their final approval of the version to be published.
